# Primary myelofibrosis associated glomerulopathy: significant improvement after therapy with ruxolitinib

**DOI:** 10.1186/s12882-015-0121-6

**Published:** 2015-08-01

**Authors:** Arun Rajasekaran, Thuy-Trang Ngo, Maen Abdelrahim, William Glass, Amber Podoll, Srdan Verstovsek, Ala Abudayyeh

**Affiliations:** Department of Internal Medicine, The University of Texas MD Anderson Cancer Center, Houston, TX USA; Department of Nephrology, The University of Texas Medical School at Houston, Houston, TX USA; Division of Medical Oncology, Duke University School of Medicine, Durham, NC USA; Department of Renal Pathology, The University of Texas Medical School at Houston, Houston, TX USA; Department of Leukemia, The University of Texas MD Anderson Cancer Center, Houston, TX USA; Division of Internal Medicine, Section of Nephrology, The University of Texas MD Anderson Cancer Center, 1515 Holcombe Blvd., Houston, TX 77030 USA

**Keywords:** Myelofibrosis, Glomerulopathy, Proteinuria, Acute kidney injury

## Abstract

**Background:**

Primary myelofibrosis (PMF) is a type of myeloproliferative neoplasm (MPN) characterized by the predominant proliferation of megakaryocytes and granulocytes in the bone marrow, leading to the deposition of fibrous tissue, and by a propensity toward extramedullary hematopoiesis. Renal involvement in PMF is rare, but kidney tissue samples from these patients reveal MPN-related glomerulopathy, a recently discovered condition, in the late stages of the disease.

**Case presentation:**

We present the first case described in the medical literature of a patient with early renal glomerular involvement in PMF/MPN. A 60-year-old man with stage 4 chronic kidney disease and a recent diagnosis of PMF (within 4 weeks of presentation at our renal division) presented with generalized body swelling, acute kidney injury, and massive nephrotic-range proteinuria. Kidney biopsy was performed to determine the etiology of the patient’s renal dysfunction and revealed early renal glomerular involvement that was histologically characteristic of MPN-related glomerulopathy. Early diagnosis and prompt medical management returned the patient’s kidney functionality to the levels seen on initial presentation at our hospital.

**Conclusion:**

Large studies with long follow-up durations are necessary to identify and categorize the risk factors for the development of MPN-related glomerulopathy, to standardize therapeutic regimens, and to determine whether aggressive management of the myelofibrosis slows the progression of kidney disease.

## Background

Myeloproliferative neoplasms (MPNs) are clonal hematopoietic stem cell disorders in which proliferation of one or more of the myeloid lineages occurs owing to acquired somatic mutations in signal transduction pathways, which results in fibrosis of the bone marrow. MPNs generally occur in the elderly [1]. Primary myelofibrosis (PMF), a type of MPN, is defined by the predominant proliferation of megakaryocytes and granulocytes in the bone marrow, eventually leading to the deposition of fibrous tissue with progressive pancytopenia, and by a propensity toward extramedullary hematopoiesis, including enlargement of the liver and spleen [2]. The major complications associated with MPN disease include increased risk of thrombosis and hemorrhage and transformation into acute myeloid leukemia [3]. Renal involvement in MPN is infrequent. Acute kidney injury may develop because thrombosis of the renal vessels , occlusion of the urinary tract by blood clots, tumor lysis syndrome, or due to leukemic infiltration of the interstitium [4, 5].

Patients with MPN develop a distinct glomerular lesion, recently described as MPN-related glomerulopathy, which differs in morphology and pathology from those caused by other hematologic neoplasms [4, 6]. MPN-related glomerulopathy is the first glomerular injury that has been associated with myeloid neoplasms [7]. Typical clinical presentation includes nephrotic-range proteinuria (with or without the full nephrotic syndrome) and chronic renal insufficiency. Diagnosis is confirmed by kidney biopsy; tissue samples show the histopathological pattern associated with MPN-related glomerulopathy comprising variaing degrees of mesangial hypercellularity and sclerosis, segmental sclerosis, and intracapillary hematopoietic cell infiltration (most commonly with megakaryocytes) on light microscopy and absence of immune deposits on immunofluorescence and electron microscopy. Additionally, segmental subendothelial electron-lucent thickening with glomerular basement membrane double contours is seen in some patients, mimicking a chronic thrombotic microangiopathy [7].

Proper recognition of MPN-related glomerulopathy and differentiation of this renal disorder from other forms of sclerosing glomerulopathy, thrombotic microangiopathy, and immune-complex glomerulonephritis are essential for diagnosis and management. We describe the first case in the medical literature of an early manifestation of MPN-related glomerulopathy in a patient recently diagnosed with PMF, and significant improvement in kidney function upon successful therapy of PMF with ruxolitinib, an oral JAK inhibitor.

## Case report

A 60-year-old white man with stage 4 chronic kidney disease (CKD), coronary artery disease, hypertension, dyslipidemia, and a recent diagnosis of PMF presented at our renal division with generalized body swelling and an elevated serum creatinine level with massive nephrotic-range proteinuria. His serum creatinine level was 1.78 mg/dL at the initial consultation at our institution. He was a former smoker of 35 pack-years.

The examination revealed diminished bibasilar breath sounds and severe abdominal distension with massive hepatosplenomegaly along with profound anasarca. No skin rashes were seen. Laboratory work-up revealed thrombocytosis, hyperlipidemia, and hypoalbuminemia. Urinalysis revealed proteinuria at 600 mg/dL and 300 mg/dL of glucose, and the spot urine sample had a protein to creatinine ratio of 23 along with multiple granular casts. The estimated glomerular filtration rate was 27 mL/min/1.73 m^2^ (normal range, 90–120 mL/min/1.73 m^2^).

Given his massive proteinuria, hypoalbuminemia, and peripheral edema, the differential diagnoses included focal segmental glomerulosclerosis (secondary to myelofibrosis), membranous glomerulonephritis, amyloidosis, and paraneoplastic glomerulonephritis. Results from the proteinuria assessment and work-up with serum and urine protein electrophoresis, immunofixation, hepatitis and human immunodeficiency virus tests, and tests for antinuclear antibodies and other immunological markers were unremarkable. Transthoracic echocardiography images showed no valvular abnormalities, and the patient had an ejection fraction of 60 % (normal value > 55 %).

The patient was placed on a salt and fluid restricted diet and was treated with bumetanide, lisinopril, hydralazine, carvedilol, isosorbide mononitrate, and rosuvastatin. He was on hydroxyurea for PMF. He underwent a kidney biopsy (less than 30 days from his initial diagnosis of PMF and nephrotic syndrome) to determine the etiology of his massive proteinuria and further worsening kidney injury**.**

The patient’s symptoms improved with treatment over the next 2 weeks, with a reduction in body swelling and abdominal distension. Therapy with a new JAK inhibitor (ruxolitinib) was started; after 4 months of treatment, the patient’s white blood cell count decreased from 61,000 K/ul to 12,000 K/ul and platelets from 1152,000 K/ul, to normal levels, decrease in splenomegaly from 13 cm to 4 cm, and hepatomegaly from 14 cm to non-palpable,. This was accompanied with a decrease in his protein to creatinine ratio to 8 g from 23 g and return of his baseline creatinine to 1.78 mg/dL from a peak value of 4.23 mg/dL.

## Kidney biopsy

**Fig. 1:**
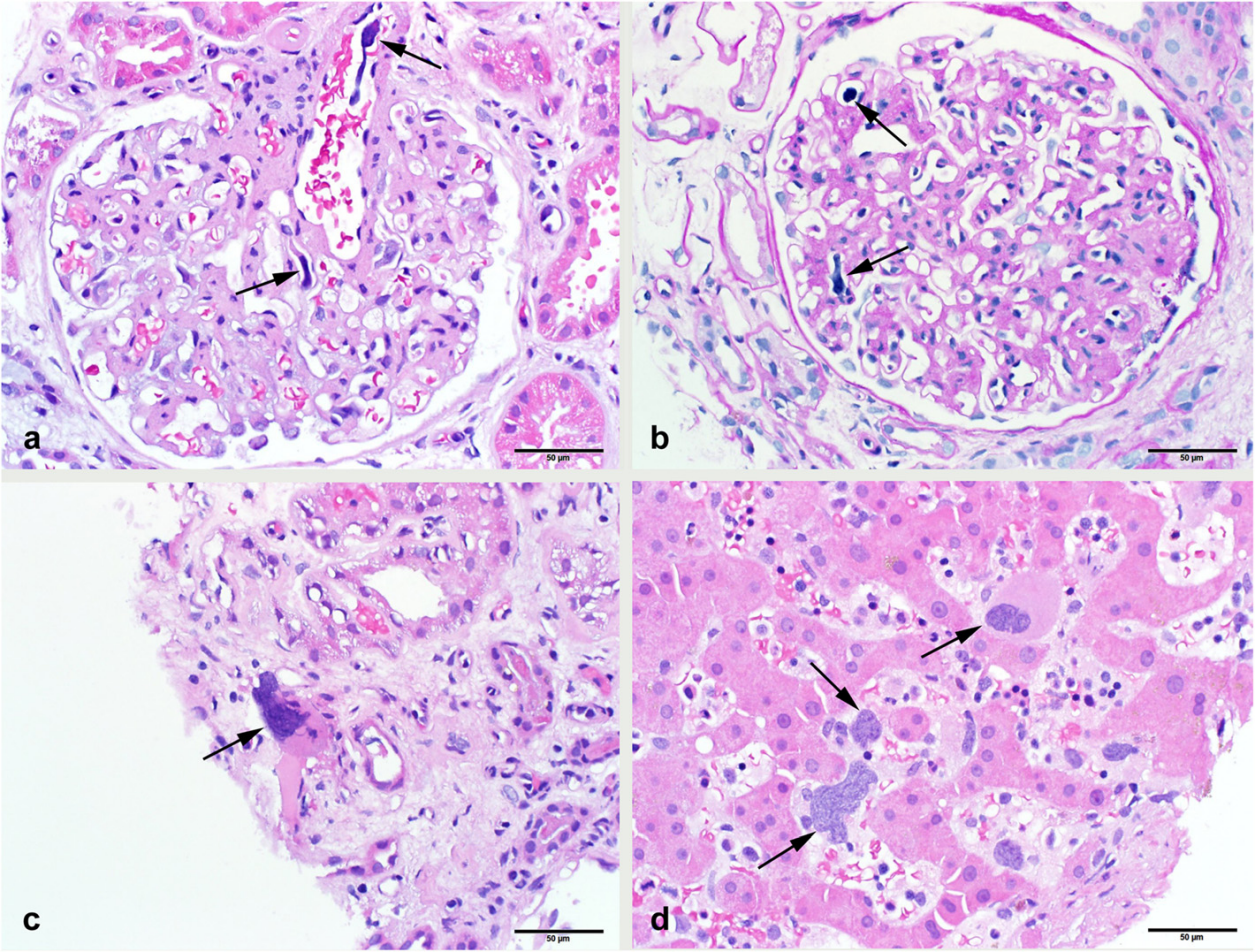
Microscopy results of kidney and liver tissue from a 60-year-old man with myeloproliferative neoplasm-related glomerulopathy in the early stages of primary myelofibrosis hematoxylin and eosin (H&E) (**a**) and periodic acid-Schiff (PAS) (**b**) stained sections of glomeruli with moderate mesangial matrix expansion and occasional intracapillary and intra-arteriolar megakaryocytes (arrows). The renal tubulointerstitium (**c**) also contained interstitial or intracapillary megakaryocytes (arrow). A biopsy of the liver (**d**) showed megakaryocytes, nucleated red blood cells and other immature myeloid cell within the hepatic sinusoids

**Fig. 2:**
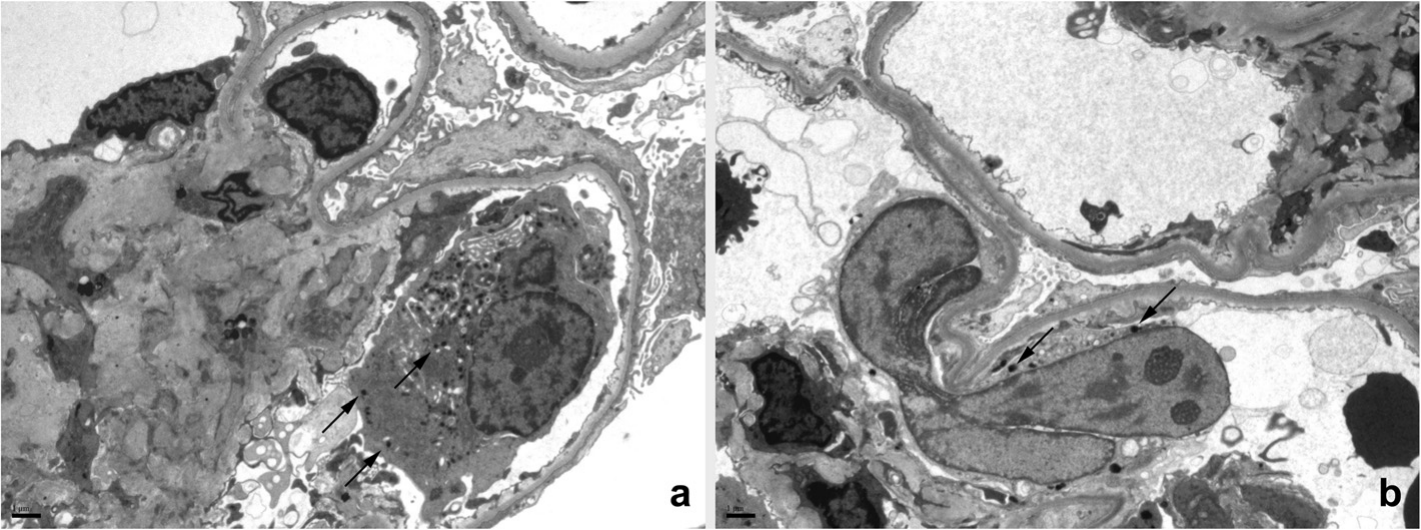
Electron micrographs show glomeruli with segmental intracapillary megakaryocytes (a and b) and mesangial matrix expansion (**a**). Podocyte foot processes were extensively effaced. Megakaryocytes contained characteristic small dense core granules (arrows) and multilobular nuclei (**b**)

Light microscopic examination of the kidney tissue (Fig. 1a-c) revealed twelve glomeruli which were slightly enlarged with diffuse mesangial matrix expansion and mild mesangial hypercellularity; however, the segmental capillaries also contained cells with large atypical-appearing nuclei consistent with megakaryocytes. The expanded mesangial matrix stained positive for periodic acid-Schiff and Grocott methenamine silver. One glomerulus was globally sclerotic and no glomerulus was segmentally sclerotic. There was a diffuse mild to moderate increase in tubulointerstitial matrix, but without tubular atrophy. Only a scant mononuclear inflammatory infiltrate was present. Tubules were mildly ectatic focally with loss of brush borders. Interstitial capillaries also contained occasional megakaryocytes. Mild to moderate arteriosclerosis with focal hyaline arteriolar sclerosis was present. Immunofluorescence showed weak to 1+ mesangial IgM, but glomeruli were negative for IgG, IgA, kappa light chain, lambda light chain, C3 and C1q. Electron microscopic examination of three glomeruli (Fig. 2) revealed diffuse, moderate expansion of the mesangial matrix with very focal segmental deposits of slightly electron- dense material, but typical immune complex deposits were not present. Occasional megakaryocytes with characteristic small dense core granules and immature myeloid-series cells were seen within the capillaries. Podocyte foot processes were extensively effaced.

### Liver biopsy

A liver biopsy, performed at the same time as the kidney biopsy (Fig. 1d) showed extramedullary hematopoiesis characterized by dilated sinusoids which contained moderate amount of immature myeloid cells including megakaryocytes, nucleated red blood cells, and myelocytes in various stages of maturation and differentiation.

## Discussion

The morphological differential diagnosis of MPN-related glomerulopathy includes diabetic glomerulosclerosis, smoking-related glomerulopathy, primary focal segmental glomerulosclerosis, thrombotic microangiopathy, and chronic membranoproliferative glomerulonephritis. Mesangial hypercellularity is more prominent in MPN-related glomerulopathy than in diabetic glomerulosclerosis, smoking-related glomerulopathy, and primary focal segmental glomerulosclerosis, whereas nodular mesangial sclerosis is not typically associated with MPN-related glomerulopathy. Absence of immune deposits revealed by immunofluorescence and electron microscopy differentiates MPN-related glomerulopathy from membranoproliferative glomerulonephritis [7]. Though it is well known that the myeloproliferation in MPN results from a clonal expansion of the myeloid progenitor cells, the subsequent myelofibrosis definitive of PMF is hypothesized to be due to a reactive mechanism mediated by megakaryocyte-derived excessive synthesis of growth factors including platelet-derived growth factor and transforming growth factor-β [8, 9]. Platelet-derived growth factor stimulates mesangial cell proliferation, mesangial matrix synthesis, and apoptosis of podocytes, whereas transforming growth factor-β aids in mesangial cell production of collagen and fibronectin. The combination of these effects could cause glomerular lesions specific to MPN-related nephropathy. Furthermore, aggregation of circulating hematopoietic cells within glomerular presumably capillaries could presumably lead to endothelial injury and morphological changes mimicking chronic thrombotic microangiopathy [10, 11].

In a recent series of 11 patients with MPN who developed proteinuria and renal insufficiency, most of the patients (73 %) had PMF, a less prevalent type of MPN, suggesting that patients with PMF have a higher risk of developing MPN-related glomerulopathy than do patients with other types of MPN [7]. However, PMF is not considered a pure paraneoplastic disease because of the presence of hematopoietic cell infiltration. In these 11 patients, the clinical manifestations of MPN-related glomerulopathy, namely proteinuria of greater than 3 g/day with chronic renal insufficiency, tended to manifest late in the course of MPN (the mean time from diagnosis of MPN to kidney biopsy was 7.2 years) [7].

In a recent study, the frequency of CKD in a large cohort of 143 MPN patients from Denmark at time of diagnosis was 29 %: 27 % had stage 3 CKD, and 2 % had stage 4 CKD. This was the first study to describe the progression patterns in renal function over time in MPN patients [12]. This important and speculative finding of a high frequency of CKD at diagnosis of MPN suggests that MPN disease detrimentally affects kidney function. In this context, MPN and CKD could be linked by chronic inflammation, which is hypothesized to be a trigger and amplifier of the MPN disease process [13].

The prognosis of patients with MPN-related glomerulopathy, unlike those with other glomerulopathies, remains guarded even after corticosteroid therapy, renin-angiotensin system blockade, and treatment of the underlying neoplasm [7]. Most patients continue to have persistent renal dysfunction with progression to end-stage renal disease, and therefore it was very satisfying to witness significant clinical improvement in patient’s condition and renal function on therapy with JAK inhibitor, ruxolitinib. Because PMF and MPN-related glomerulopathy were detected within a relatively short time in our patient, prompt medical management restored stable renal function. However, because our patient also has stage 4 CKD and multiple co-morbidities, his long-term prognosis remains poor.

## Conclusions

Larger studies with longer follow-up durations are necessary to identify and categorize the risk factors for the development of MPN-related glomerulopathy, to standardize therapeutic regimens, and to determine whether aggressive management of the myelofibrosis slows the progression of kidney disease. We recommend that patients with hematological neoplasms should be screened for urinary abnormalities, especially proteinuria. An aggressive approach to urinary screening and kidney biopsy would provide more data on the frequency and course of renal disease in MPN and would provide key information on the feasibility of conducting clinical trials for treating MPN-related glomerulopathy.

## Consent

Written informed consent was obtained from the patient for publication of this Case report and any accompanying images. A copy of the written consent is available for review by the Editor of this journal.

## Abbreviations

CKD: Chronic Kidney Disease
MPN: Myeloproliferative Neoplasm
PMF: Primary Myelofibrosis
